# Navigating cognitive dissonance: master’s students’ experiences with ChatGPT in dissertation writing

**DOI:** 10.3389/fpsyg.2025.1542559

**Published:** 2025-05-09

**Authors:** Xiaobin Ren, Wenwen Zheng, Min Zhang

**Affiliations:** School of Foreign Languages, Guangxi University, Nanning, China

**Keywords:** cognitive dissonance, dissertation writing, ChatGPT, grounded theory, navigating strategies

## Abstract

With the increasing prevalence of AI tools like ChatGPT in academic settings, understanding their impact on students’ psychological experiences during dissertation writing is crucial. This study aims to explore the cognitive dissonance experienced by master’s students during dissertation writing with the assistance of ChatGPT and identify the strategies they employ to manage this dissonance. Using grounded theory as the primary research methodology, we analyzed 28 interview transcripts to uncover key elements of cognitive dissonance and develop a corresponding theoretical model. Our findings revealed that the primary sources of cognitive dissonance among master’s students were the strong intentions to use ChatGPT driven by subjective norms and technological expectations, conflicting with the reality of multiple choices. To alleviate this cognitive dissonance, students adopted strategies such as improving prompt quality, feeding relevant domain-specific data to the AI, avoiding academic misconduct, and maintaining academic integrity. This study challenged and extended the Technology Acceptance Model and the Theory of Planned Behavior by incorporating cognitive dissonance, and emphasized the underlying pathways and causes of dissonance. Practically, our findings offer significant implications for institutions and educators, emphasizing the importance of supporting and guiding the use of generative AI tools like ChatGPT in dissertation writing.

## 1 Introduction

The rapid advancement of artificial intelligence (AI), particularly tools like ChatGPT, has significantly impacted various fields, including education (Adeshola and Adepoju, 2023; Kieser et al., 2023; Ye et al., 2024) and academic research (Chatterjee et al., 2023; Rahman and Watanobe, 2023). In educational settings, AI tools are increasingly used by students at various levels to enhance productivity and streamline the writing process. However, for graduate students, particularly those pursuing master’s degrees, the stakes are notably higher compared to undergraduate or doctoral students. Master’s students, often at a transitional stage of developing independent research skills, are required to produce original work that bridges foundational knowledge with more advanced critical thinking and academic writing abilities (Zhou et al., 2022). Unlike doctoral students, who have more experience in research, or undergraduates, who are generally focused on coursework, master’s students face unique pressures to balance learning research methodologies with demonstrating independent scholarly contributions. While ChatGPT can assist in this process, over-reliance on such tools may undermine the development of these essential competencies (Fuchs, 2023). Furthermore, the blurred boundaries between AI-assisted writing and plagiarism introduce ethical dilemmas for both students and institutions (Cotton et al., 2023). In the case of Chinese master’s students, these challenges are exacerbated by the legal and ethical constraints on accessing ChatGPT due to restrictions in China (Hung and Chen, 2023), creating heightened psychological tension as they navigate the academic benefits of using the tool and the potential consequences of bypassing such restrictions (Liu et al., 2024), a tension which reflects a core manifestation of cognitive dissonance—defined as the psychological discomfort resulting from conflicting beliefs, attitudes, or behaviors (Festinger, 1957).

Although cognitive dissonance has been widely explored in fields such as marketing, management, and consumer behavior (Karanika-Murray et al., 2017; Marikyan et al., 2023; Wilkins et al., 2016), its application in educational contexts remains limited. This gap is particularly important to address, given that high-stakes academic settings like thesis writing are often accompanied by internal conflict and psychological tension, which can shape students’ academic choices and emotional states (Collier and Rosch, 2016). In the context of AI-assisted writing, such dissonance may occur when students rely on tools like ChatGPT to enhance their theses while simultaneously perceiving this behavior as misaligned with academic norms or personal values. Therefore, understanding how students manage this internal conflict is critical for informing supportive educational strategies and promoting ethical, balanced technology use.

In addition, recent research on academic writing has largely focused on the use of AI tools like ChatGPT, assessing their benefits and potential impact on students’ skill development (Lingard, 2023). These studies have also explored the broader psychological effects of technology in education (Al-Takhayneh et al., 2022) and ethical concerns surrounding AI usage (Alasadi and Baiz, 2023). However, these investigations primarily address general technology use and Western perspectives on AI ethics and legality. There is a notable lack of research examining the cognitive dissonance experienced by students during high-stakes writing tasks. This is particularly relevant for master’s students in regions like China, where restrictive internet policies add an additional layer of complexity to the use of AI tools. Given the unique legal and infrastructural restrictions on ChatGPT access in China, the cognitive dissonance observed in this study may reflect context-specific tensions not equally present in other regions.

This study investigated the cognitive dissonance experienced by master’s students in China using ChatGPT in their thesis writing. Through semi-structured interviews and grounded theory methodology, we explored their experiences and coping strategies, aiming to identify effective ways to alleviate this dissonance. By addressing these challenges, we can develop targeted interventions and support systems that enhance students’ academic writing processes, improve their academic performance, and promote psychological well-being. The findings of this research have the potential to inform educational policies and practices, ensuring a balanced and ethical integration of AI technologies in academic settings while maintaining students’ cognitive consistency.

## 2 Literature review

### 2.1 Studies on cognitive dissonance

Cognitive dissonance theory, introduced by Festinger (1957), refers to the psychological discomfort individuals experience when they hold contradictory beliefs, values, or attitudes, or when their behavior is inconsistent with their beliefs. In such situations, individuals are motivated to reduce this discomfort through changes in cognition, behavior, or the reinterpretation of information.

Over the past decades, cognitive dissonance theory has been widely applied across fields such as marketing and consumer behavior (Wilkins et al., 2016), environmental psychology (Mi et al., 2019; Yang et al., 2024), and organizational studies (Karanika-Murray et al., 2017). Studies have shown that dissonance can lead to emotional reactions such as guilt, regret, or frustration (Marikyan et al., 2023), and may trigger behavioral adjustments to restore internal consistency (McGrath, 2017). Despite receiving less attention than in other disciplines, cognitive dissonance has begun to attract scholarly interest in education, where students often encounter conflicting expectations, learning goals, and institutional pressures. Emerging studies have examined its role in learning behaviors (Atoum and Al-Adamat, 2024), and achievement motivation (Saveh, 2018), indicating that students frequently experience psychological discomfort when navigating misalignments between internal values and academic demands. However, many important learning contexts remain underexplored, and further research is needed to understand how students experience and respond to dissonance in complex academic environments.

Among the underexplored areas within educational research, academic writing—particularly high-stakes tasks such as master’s dissertation writing (Carter and Kumar, 2017)—has received even limited attention through the lens of cognitive dissonance theory. Master’s students are often required to produce original, high-quality work while simultaneously managing self-doubt, institutional expectations, and time pressure (Trimble et al., 2025). These conflicting demands can create a unique form of cognitive dissonance, particularly when students rely on new technologies such as ChatGPT. While existing research highlights the psychological and emotional impact of dissonance (Kenworthy et al., 2014), little is known about how it emerges in AI-assisted writing contexts, or what strategies students use to manage it.

### 2.2 AI-assisted academic writing

Graduate-level academic writing is often a cognitively and linguistically demanding process, particularly for non-native English-speaking students who are required to produce high-quality research outputs in English (Aldabbus and Almansouri, 2022; Qadir et al., 2021). Traditional solutions—such as translation tools or professional editing services—have shown limited effectiveness or accessibility (Tongpoon-Patanasorn, 2020). The emergence of generative AI tools like ChatGPT has introduced new possibilities by offering real-time feedback, improving linguistic expression, and reducing students’ cognitive load (Hwang et al., 2023; Kayaalp et al., 2024). Empirical studies have confirmed the tool’s utility in enhancing writing productivity, especially among postgraduate students (Bouzar et al., 2024), and in supporting English academic writing for non-native researchers (Hwang et al., 2023).

However, recent literature also highlights a growing set of ethical, pedagogical, and psychological concerns associated with AI-assisted writing. Students may become over-reliant on AI-generated text (Wang et al., 2023; Ye et al., 2025), struggle to preserve their academic voice and originality (Koos and Wachsmann, 2023), or feel uncertain about the acceptability of using AI under institutional policies (Mondal and Mondal, 2023). These concerns are often heightened in high-stakes academic contexts, such as thesis writing, where the boundaries between legitimate assistance and academic misconduct are frequently ambiguous (Cotton et al., 2023).

As several scholars point out, while ChatGPT can assist with linguistic and structural elements of academic writing, it cannot replace human judgment, critical thinking, and intellectual ownership (Lo, 2023; Mondal and Mondal, 2023). Despite these important insights, current research remains largely focused on evaluating the technical capabilities or ethical boundaries of ChatGPT use. There is a lack of in-depth exploration into the cognitive and psychological tensions students experience when using this AI tool (Hong et al., 2024; Ye et al., 2023). Specifically, little is known about how master’s students navigate conflicting cognitions—such as valuing academic integrity while relying on AI support—and what strategies they employ to manage these internal conflicts. This presents a critical research gap, as understanding these psychological processes is essential for developing informed educational policies, responsible AI use guidelines, and practical academic writing support systems.

To address this need, our study focuses on two main research questions:

1) What specific cognitive dissonance processes do master’s students experience when using ChatGPT in thesis writing?
2) What strategies do students employ to manage cognitive dissonance while using ChatGPT?

By investigating these questions, we aim to provide a comprehensive understanding of the interplay between AI tools and cognitive dissonance in academic writing, ultimately contributing to the development of more effective educational interventions and policies.

## 3 Research design

### 3.1 Research methodology

This study employs Grounded Theory (Strauss and Corbin, 1994) as the primary research method. Grounded Theory is a systematic methodology in the social sciences that involves the collection and analysis of data to construct theories. Initially proposed by sociologists Glaser and Strauss (1967), it is particularly suitable for exploring unknown phenomena and aims to create theoretically sound models that explain how participants manage issues and processes in their daily lives. Its iterative process of data collection and analysis (Orton, 1997) distinguishes it from other methods, making it ideal for investigating complex psychological phenomena such as cognitive dissonance.

This methodology allows theories to emerge from the data (Corbin and Strauss, 2014), ensuring that the findings are deeply rooted in the actual experiences of the participants. Through the cyclical process of data collection, coding, and analysis, Grounded Theory enables researchers to adjust the research direction flexibly based on the findings in the field. This flexibility is crucial for capturing the nuanced ways in which master’s students may experience cognitive dissonance during the dissertation writing process.

### 3.2 Research context

In universities in mainland China, graduate students are required to write a master’s thesis to obtain a master’s degree. The thesis evaluation process is high-stakes, requiring a score of 70 (out of 100) or above from two external reviewers for students to qualify for the defense. Failure to meet this standard directly impacts their graduation prospects. For students in English-related majors, such as Translation Studies, English Language and Literature, Foreign Linguistics and Applied Linguistics, Area Studies, and the Master of Translation and Interpreting (MTI) program, the stakes are even higher as they are typically required to write their theses in English. The requirement to produce a high-quality thesis in a foreign language adds an additional layer of complexity to their academic journey.

ChatGPT, a generative AI tool that has gained global popularity, is widely accepted for its exceptional language capabilities, particularly among English-related graduate students. However, since OpenAI has not yet made ChatGPT available in mainland China, graduate students face numerous technical challenges when attempting to use this advanced language generation model. These challenges include accessing the tool through VPNs, which is both technically demanding and legally ambiguous, and concerns over data privacy and security. Additionally, the lack of localized support and guidance on using such tools in an academic context further complicates their effective use.

### 3.3 Sampling method

This study followed the principles of grounded theory and employed theoretical sampling (Glaser and Strauss, 2006) to guide the selection of participants. Theoretical sampling is a data collection method in qualitative research that is driven by concepts emerging from previously collected data. It allows for the development and refinement of theory as more data is collected and analyzed, focusing on areas that require further exploration to build a robust theoretical framework. In addition to adhering to the principles of theoretical sampling, participants in this study had to meet the following criteria: they must be current master’s students or recent graduates within the last 6 months; participants should have used ChatGPT extensively during the drafting and revision stages of their theses.

Based on the above standards and criteria, this study selected 28 master’s students from English-related majors (all of whom wrote their master’s theses in English) across three universities in China before the data reached saturation. All of the three universities have the authority to confer master’s degrees in foreign languages and literature. These universities, chosen for their diversity in type (comprehensive, technical, and normal) and geographical location, provided a broad range of academic environments and regional perspectives. This selection ensured a representative sample of students with varied experiences using ChatGPT in thesis writing. The specific details of the participants are shown in Table 1.

**Table 1:** Participants’ information.

Participant ID	Gender	Age	Major/Research area
P01	F	24	Translation-Academic
P02	F	25	Linguistics
P03	F	26	Area studies
P04	F	26	Linguistics
P05	M	25	Linguistics
P06	F	25	Translation-Academic
P07	F	24	English literature
P08	F	26	Linguistics
P09	F	25	Area studies
P10	F	26	Translation-Professional
P11	F	28	Translation-Professional
P12	F	26	Area studies
P13	F	25	English literature
P14	F	24	Translation-Academic
P15	M	26	English literature
P16	F	26	Linguistics
P17	F	25	Area studies
P18	F	24	English literature
P19	F	26	Linguistics
P20	F	25	Area studies
P21	F	25	Linguistics
P22	F	25	Translation-Professional
P23	M	26	Translation-Academic
P24	F	29	Area studies
P25	F	28	Translation-Professional
P26	M	25	English literature
P27	F	26	Translation-Academic
P28	F	27	Linguistics

### 3.4 Instruments and data collection

This study employed semi-structured interviews for data collection. To ensure the validity of the semi-structured interviews, an interview outline was initially drafted, and two foreign language teaching experts with doctoral degrees were invited to validate the outline and provide suggestions for revisions. The final interview outline mainly focused on the experiences, cognitions, and perceptions of master’s students using ChatGPT to assist in writing their theses. The questions in the interview outline included: “Can you describe at what stage of your thesis writing you started using ChatGPT?,” “Did you experience any discomfort or conflict while using ChatGPT in your thesis writing?,” “How did you feel about the information or assistance provided by ChatGPT in your thesis writing?,” “How did you resolve or cope with the discomfort or conflict feelings experienced while using ChatGPT?” and other related questions.

Before interviewing the 28 master’s students, the purpose of the interview and the data usage method were explained, with a promise of data confidentiality and proper usage. The audio recordings of all the interviews with the graduate students were obtained with their informed consent. The interviews with each participant lasted an average of 35 min.

### 3.5 Data analysis

This study is based on Strauss and Corbin (1994) grounded theory to organize and analyze the collected interview data. To enhance coding efficiency, all interview transcripts were imported into NVivo14 for coding analysis. First, 21 interview transcripts (3/4 of the total) were randomly selected from the 28 collected for open coding, axial coding, and selective coding. Through these steps, concepts were continuously refined and categorized, and the logical elements of cognitive dissonance experienced by master’s students using ChatGPT in their thesis writing process were summarized. This process led to the construction of a theoretical model reflecting the cognitive dissonance of master’s students. Finally, the remaining 7 interview transcripts (1/4 of the total) were used to test the theoretical saturation and further refine and develop the theoretical model.

#### 3.5.1 Open coding

Open coding is the process of meticulously analyzing and summarizing data to present raw material as a series of significant concepts and categories (Strauss and Corbin, 1994). Through in-depth analysis of the policy texts, 72 initial concepts were generated, which were further refined into 16 subcategories. Partial results of the open coding are shown in Table 2. For instance, one participant noted, “Our school’s academic affairs office even issued specific guidelines and precautions for students using large language models.” This statement was coded as “School guidance policy,” reflecting the institutional support provided to students regarding the use of AI tools like ChatGPT. This initial concept was then refined into the subcategory “Institutional regulation,” highlighting the broader regulatory framework guiding students’ interactions with emerging technologies in academic contexts. This example illustrates how we systematically extracted meaningful insights from the data, forming the foundation for subsequent analyses.

**Table 2:** Open coding (excerpt).

Original data	Initial concepts	Subcategories
When ChatGPT first came out, I saw endless introductions to this tool on TikTok.	Introduction on video platforms	Promotional effect
Our advisor often mentioned in group meetings that we should use it for language polishing.	Advisor recommends use	Supervisor support
Our school’s academic affairs office even issued specific guidelines and precautions for students using large language models.	School guidance policy	Institutional regulation
If it does not answer well, do you ever reflect on your instructions? Maybe they were not detailed enough, or not specific enough, or lacked direction.	Detail and specificity of prompts	Prompt quality
For instance, I can have it read 50 recent relevant articles first.	Have ChatGPT read articles	Feeding the model
Because it only scrapes some established online corpora and generates content it deems suitable according to your instructions, but verifying whether the content is appropriate or true is still up to humans.	Content verification	Avoiding academic misconduct
The underlying viewpoints are mine. Even if I use ChatGPT to assist me in writing, it does not involve academic integrity issues.	Ensuring originality of views	Integrity awareness
There are many culturally loaded words that it does not understand.	Limited cognitive ability	Capability deficit
Using ChatGPT also requires bypassing internet restrictions, which is quite difficult for me.	Internet restrictions	Technical barriers
Because a lot of my content is related to Chinese culture, ChatGPT does not understand it well. Then I stopped using it.	Poor performance leading to discontinuation	Discontinuation intention
I think, for me, the domestic platforms are sufficient for my personal needs.	Acceptance of domestic models	Model competition
It might withhold my personal information.	Information leakage	Trust risk
Actually, I think most of our classmates used ChatGPT for assistance, especially in translation, but they do not disclose this in their papers.	Non-disclosure of usage	Presentation dilemma
Blind reviewers tend to raise their standards unconsciously for papers that used ChatGPT.	Controversy over usage	Evaluation confusion
By the end, there was no time left. It was too rushed, so I sought help from ChatGPT.	Efficiency improvement	Academic capability
This is an inevitable trend. It’s not about whether you want it or not. Instead, you must adapt to it.	Adapting to trends	Positive trend

#### 3.5.2 Axial coding

Axial coding is the process of further refining the numerous subcategories formed during open coding, with the goal of obtaining main categories with stronger generalization or higher levels of abstraction (Strauss and Corbin, 1994). The primary task of axial coding is to discover and construct various relationships among main categories, including causal relationships, temporal relationships, sequential relationships, contextual relationships, and similarity relationships, thereby organically linking the subcategories obtained in the previous stage. For example, a sequential relationship was identified between “Discontinuation Intention” and “Model Competition.” This relationship illustrates how master’s students may consider discontinuing their use of ChatGPT for thesis writing when faced with competitive alternatives, such as other generative AI models. In this study, axial coding was carried out based on the 16 subcategories formed during open coding. This process further summarized and refined these categories, resulting in 6 main categories. Specific information is provided in Table 3.

**Table 3:** Axial coding.

Main categories	Subcategories	Subcategory connotations
Subjective norms	Promotional effect	Various media promotions about ChatGPT stimulate master’s students to explore and use the tool.
	Supervisor support	Master’s supervisors affirm and assist students in using ChatGPT in their dissertations.
	Institutional regulation	Universities have established specific regulatory measures for using generative AI in thesis work.
Usage strategies	Prompt quality	Master’s students need to write specific and targeted prompts when using ChatGPT for thesis assistance.
	Feed the model	Master’s students can provide ChatGPT with relevant studies in advance to help it understand the latest research trends.
	Avoid academic misconduct	Master’s students should discern the accuracy and truthfulness of the information provided by ChatGPT.
	Integrity awareness	Master’s students should adhere to academic integrity while using ChatGPT.
Low Tool Efficacy	Capability deficit	ChatGPT has shortcomings in assisting with master’s thesis writing.
	Technical barriers	Master’s students face technical challenges and difficulties when using ChatGPT for thesis assistance.
Diverse options	Discontinuation intention	Master’s students consider discontinuing the use of ChatGPT for thesis writing.
	Model competition	ChatGPT faces competition from various domestic and international generative AI models.
Perceived risk	Trust risk	Master’s students believe that texts produced by ChatGPT are not entirely trustworthy.
	Presentation dilemma	Master’s students struggle to decide whether to clearly disclose their use of ChatGPT in their theses.
	Evaluation confusion	The academic community has not reached a consensus on whether master’s students can use ChatGPT in their dissertations.
Technological expectancy	Academic capability	ChatGPT can provide ideas, improve efficiency, and enhance the quality of language for master’s thesis writing.
	Positive trend	The capabilities of ChatGPT in academic writing are continuously improving.

#### 3.5.3 Selective coding

**Figure 1:**
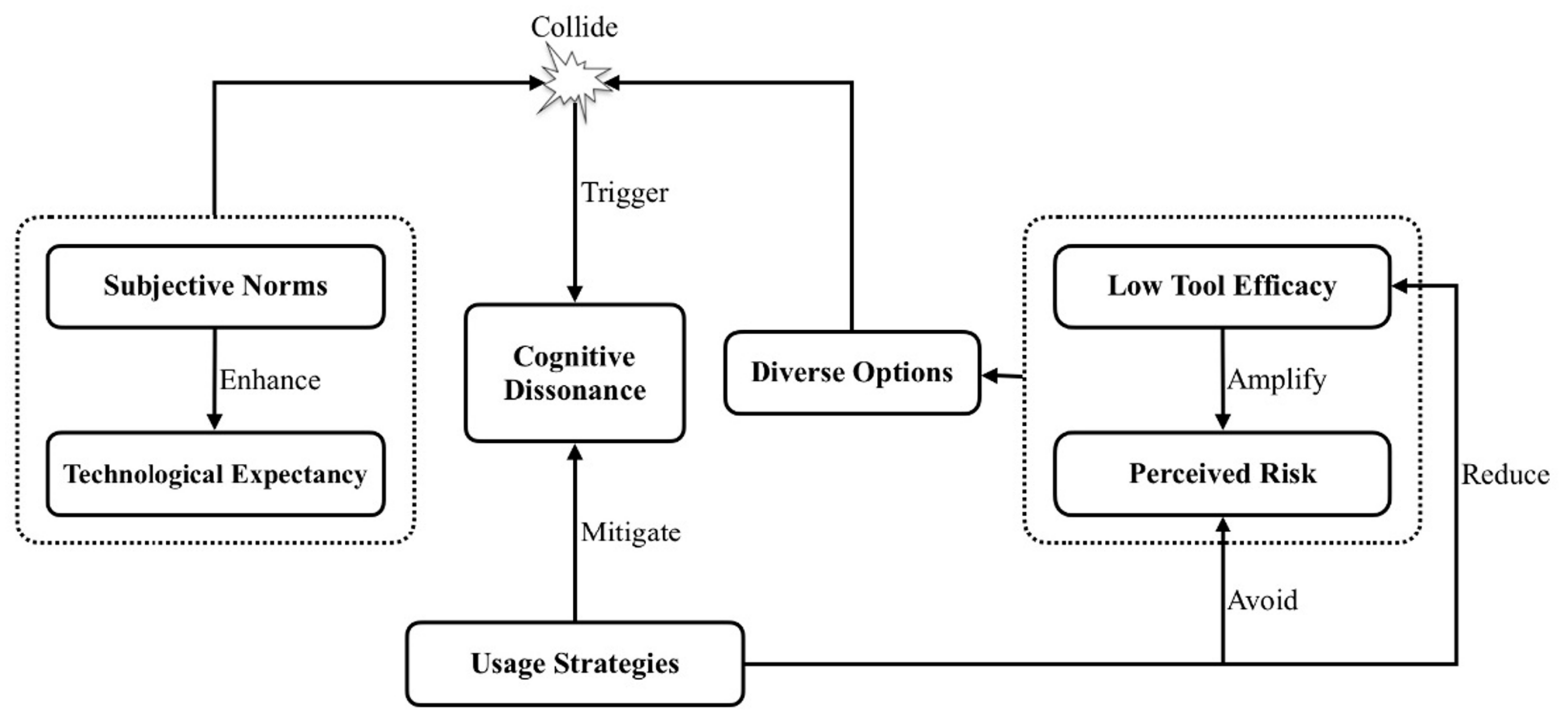
Cognitive dissonance model.

The main task of selective coding is to further analyze the multiple main categories formed during axial coding, identify a core category with greater generalization and overarching significance, and connect all other categories to this core category, thereby forming a coherent “storyline” throughout the research (Strauss and Corbin, 1994). By continuously comparing and analyzing the six main categories mentioned above, this study identified “cognitive dissonance” as the core category that links the other main categories. To further explore the logical relationships between the core category and the main categories, a “storyline” was established to illustrate these relationships, ultimately forming a typical structure of relationships among the main categories, as shown in Figure 1.

In this model, subjective norms and technological expectancy drive master’s students to use ChatGPT in thesis writing. However, this intention is challenged by low tool efficacy and perceived risk, prompting some to consider alternative options. The clash between these external pressures and usage barriers creates a cognitive dissonance. In response, students adopt various usage strategies to alleviate the discomfort.

### 3.6 Saturation test

Theoretical saturation is the criterion for stopping coding, meaning that no additional data can be obtained to allow researchers to discover new categories (Rowlands et al., 2016). In this study, by analyzing the remaining 7 interview transcripts using the same coding methods, it was found that no new concepts and categories emerged, and the relationships among categories did not change significantly. Therefore, the “Cognitive Dissonance Model for Master’s Students Using ChatGPT to Assist in Thesis Writing” constructed in this study theoretically reached saturation.

### 3.7 Triangulation for validating findings

To enhance the credibility of the findings and address potential subjectivity in student self-reports, this study incorporated a triangulation strategy by conducting semi-structured interviews with three experienced master’s thesis supervisors. All three participants held doctoral degrees, were active faculty members in foreign language-related disciplines, and had successfully supervised students through the full thesis writing and defense process. The interviews focused on the supervisors’ observations and attitudes toward the use of ChatGPT in academic writing, including whether they noticed students experiencing hesitation or psychological tension, their concerns about academic integrity, and their views on learning outcomes. Sample interview questions included: “Have you noticed any hesitation or tension among students regarding the use of ChatGPT in thesis writing?,” “In your opinion, what are the main concerns students face when using AI tools like ChatGPT?,” and “How do you view the use of such tools in terms of academic integrity and learning outcomes?” Each interview lasted approximately 38 min. The findings from these faculty interviews were consistent with the patterns identified in the student data, thereby reinforcing the validity of the theoretical model developed through grounded theory.

## 4 Research results and discussion

This study primarily explores the cognitive dissonance experienced by master’s students while using ChatGPT to assist with thesis writing and constructs a cognitive dissonance model. Through the analysis of interview data from 28 master’s students, six main categories were identified and refined: subjective norms, technical expectancy, usage strategies, low tool efficacy, diverse options, and perceived risk. Further selective coding analysis revealed “cognitive dissonance” as the core category, which governs and connects all the main categories, forming a comprehensive theoretical model. The study found that the cognitive dissonance faced by master’s students in thesis writing primarily arises from a tension between the perceived pressure or expectation to use the tool and their reluctance or hesitation to actually rely on it. To alleviate this cognitive dissonance, the students adopted various usage strategies to improve tool efficacy and mitigate academic risks.

### 4.1 External pressures and expectations

#### 4.1.1 Subjective norms

Subjective norms refer to the social pressures and support that master’s students perceive from their surroundings to drive ChatGPT usage in thesis writing. These pressures and support may come from information media, advisors, and school policies, which can drive students to actively try using ChatGPT for assistance in their writing process. Positive promotional effects, supervisor support, and clear institutional norms enhance students’ technological expectancy of ChatGPT, making them more willing to use this tool in their thesis writing. This aligns with Ajzen’s (1991) Theory of Planned Behavior (TPB), which posits that subjective norms are the social pressures individuals feel regarding whether to perform a particular behavior, reflecting the influence of significant others or groups on individual decision-making. In this study, master’s students, influenced by media promotion, advisor encouragement, and policy guidance, developed a strong desire to use ChatGPT in their thesis writing. Additionally, this study is consistent with many empirical studies, such as Al-Qaysi et al., (2024), who also found that students’ subjective norms could positively predict their ChatGPT use behaviors in their learning practice. Recent research by Ren (2025) similarly demonstrated that subjective norms played a critical role in shaping translation majors’ intentions to use ChatGPT for translation learning and practice, further underscoring the importance of social and institutional influences in AI tool adoption.

#### 4.1.2 Technological expectancy

This study found that graduate students have positive expectations regarding the use of ChatGPT in academic writing, largely due to its current robust academic capabilities and a recognition of the promising future of generative AI technology. This finding is consistent with Wu et al. (2025), who also reported that users’ performance expectancy of ChatGPT significantly influenced their intention to adopt the tool, highlighting the critical role of perceived technological capability in shaping usage behaviors. In this study, graduate students generally believe that ChatGPT has strong capabilities for thesis writing, reflecting their high perceived usefulness of ChatGPT in assisting with thesis writing, which in turn leads to a strong intention to use it. The Technology Acceptance Model (TAM) (Davis, 1989) demonstrated that users’ perceived usefulness of technology directly affects their intention to use it. Therefore, this study supports the path from perceived usefulness to intention in TAM. However, perceived usefulness in TAM model is largely based on current technological levels (Davis, 1989), whereas this study found that expectations for the future potential of current technology can also significantly influence user behavior. This study enriches and expands the concept of perceived usefulness in TAM model, suggesting that perceived usefulness should include not only the perceived usefulness of existing technology but also the perceived usefulness of that technology as it develops in the future.

According to TAM (Davis, 1989), external variables can influence users’ perceived usefulness. This aligns with the present study, where subjective norms, as an external variable, enhanced graduate students’ technological expectancy of using ChatGPT for thesis writing (covering the concept of perceived usefulness). However, the original TAM model primarily emphasizes the characteristics and features of the technology system itself (as see, Venkatesh et al., 2003, p. 476), with less exploration of factors outside the technological system. This is why there is a substantial body of research exploring the influencing factors of external factors beyond TAM (e.g., Na et al., 2022; Vorm and Combs, 2022). This also aligns with the view of Marangunić and Granić (2015), who suggest that additional variables should be incorporated into TAM. In fact, Venkatesh and Davis (2000) also introduced subjective norms into the Technology Acceptance Model and found that subjective norms significantly influence perceived usefulness, which is partially consistent with this study. Given the important role of subjective norms in technological expectancy found in this study, future research could consider integrating the TAM and TPB models to construct a comprehensive theoretical framework, thereby improving the explanatory power of ChatGPT usage intentions. Beyond individual factors like norms, more complex theoretical frameworks may be needed to explain students’ deeper motivational drivers. For instance, Ren’s (2025) study also combined TAM and TPB with constructs from Self-Determination Theory (Deci and Ryan, 1985), such as controlled and autonomous motivation, to explore the underlying mechanisms of ChatGPT adoption. This integration demonstrated strong model fit and theoretical complementarity. Future studies may likewise benefit from incorporating motivational theories to better capture the interplay of social pressure, personal agency, and competence needs in students’ decisions to use generative AI tools.

### 4.2 Barriers to usage

#### 4.2.1 Low tool efficacy

Low tool efficacy means the perceived inadequacy of ChatGPT to meet the expectations and requirements of graduate students during thesis writing. This main category is further divided into two subcategories: capability deficit and technical barriers. Both of these subcategories contribute to a reduced perception of the tool’s usefulness and ease of use, which are critical factors in technology acceptance, as described by TAM (Davis, 1989). But there is a notable overlap between the concepts of perceived ease of use in TAM and perceived behavioral control (PBC) in TPB (Ajzen, 1991), as both relate to an individual’s perception of the ease or difficulty in using a technology (Hansen et al., 2018). Since perceived ease of use in TAM can shape attitudes, it is plausible that PBC in TPB could similarly impact behavioral attitudes. However, TPB traditionally positions PBC as a variable parallel to attitude and subjective norms, each independently affecting behavioral intentions. This creates a conceptual tension, suggesting that PBC might more accurately function as a higher-order variable influencing attitude rather than as an entirely separate construct. To resolve this overlap in the process of integrating TAM and TPB, perceived ease of use may be adopted as the preferred construct within an integrated framework, positioned alongside perceived usefulness to explain attitudes toward ChatGPT use. Conversely, PBC can be excluded to avoid conceptual redundancy and to improve theoretical clarity. This adjustment not only resolves definitional ambiguities but also enhances the parsimony and explanatory coherence of the combined framework, particularly when applied to the study of AI adoption in high-stakes academic contexts.

#### 4.2.2 Perceived risk

Perceived risk primarily revolves around the various uncertainties and potential negative outcomes that graduate students face when using ChatGPT to assist with thesis writing. This study identified three key dimensions of perceived risk: trust risk, presentation dilemma, and evaluation confusion. Students questioned the originality and reliability of AI-generated content, raising concerns about inadvertent plagiarism. They also struggled with authorship ambiguity, unsure how to disclose their use of ChatGPT without risking negative evaluations or violating academic norms. Additionally, the lack of institutional consensus on AI-assisted writing contributed to uncertainty and psychological discomfort. In some cases, universities had issued ambiguous policies—such as notifying students that their theses would be screened by AIGC-detection tools—without clearly defining acceptable boundaries for AI use. Rather than offering clarity, these vague directives heightened students’ sense of uncertainty and risk, potentially intensifying their cognitive dissonance during the writing process.

This finding echoes the concerns raised by Zhang et al. (2025), who argue that the potential risks of ChatGPT use call for a critical stance toward its output—an idea directly supported by the perceived risk dimensions identified in our study. While much research has explored the importance of perceived risk on behavioral intentions, most studies have been limited to the commercial sector, examining its impact on consumer behaviors (Lavuri et al., 2022; Sohn, 2024). There is a lack of research on perceived risk in the educational sector. However, as advanced technologies are increasingly introduced into educational settings, their unknown risks cannot be overlooked (Berendt et al., 2020). Given this context, it becomes crucial to understand how perceived risk influences the acceptance and utilization of such technologies in education. To better comprehend this dynamic, future research could specifically explore the impact of perceived risk on the use of ChatGPT in educational contexts.

#### 4.2.3 Diverse options

Diverse options refer to the various options and alternatives faced by master’s students when using AI tools to assist in thesis writing. Low tool efficacy causes users to doubt ChatGPT’s utility, while perceived risk heightens concerns about potential negative outcomes from using ChatGPT. The combination of low tool efficacy and perceived risk drives users to explore more diverse options. In the context of growing competition among generative AI tools, these factors prompt users to continuously seek the best solution among numerous tools or even consider abandoning the use of generative AI tools altogether. For example, some students might replace ChatGPT with other local AIGC tools like Kimi, DeepSeek, local AIGC tools in China.

Our findings reveal some shortcomings in TAM and the TPB as well. These theories primarily focus on the positive factors that influence people to adopt and use a particular technology or engage in certain behaviors (Na et al., 2022; Vorm and Combs, 2022), but lack an in-depth exploration of why users abandon a technology or choose alternative products. Our study found that when master’s students perceive low tool efficacy or face high perceived risks with ChatGPT, they may opt to stop using it, especially in the context of increasing competition among language models. This finding complements the existing TAM and TPB frameworks by highlighting the importance of negative factors in technology acceptance and usage decisions. By revealing these factors, our research provides new perspectives for a more comprehensive understanding of user behavior, particularly for users facing similar dilemmas with technology choices. Future research could further explore the impact of these negative factors in different contexts and investigate how to enhance user acceptance and satisfaction by optimizing technology and managing perceived risks. This not only contributes to improving existing theoretical frameworks but also provides empirical evidence for technology design and promotion.

### 4.3 Use strategies

Usage strategies refer to the various optimization and avoidance tactics employed by master’s students when using ChatGPT to assist in thesis writing. In this study, the cognitive dissonance experienced by students primarily stems from the tension between external pressures and expectations on the one hand and barriers to effective usage on the other. These contradictions created psychological discomfort, as students struggled between the desire to leverage advanced AI capabilities and the fear of relying too heavily on a tool that might not meet academic standards or could lead to ethical issues.

Cognitive dissonance theory (Festinger, 1957) posits that individuals are driven to reduce discomfort caused by conflicting beliefs, either by adjusting their attitudes or changing their behavior. The findings of this study align closely with this theoretical perspective, demonstrating that when master’s students experienced cognitive dissonance during the process of using ChatGPT to assist with thesis writing, they were intrinsically motivated to reduce this discomfort. Confronted with tensions between their expectations, values, and actual experiences, students actively employed strategies to reconcile their conflicting thoughts about ChatGPT. On the one hand, they engaged in optimization strategies, such as improving the quality of prompts or feeding more relevant data into ChatGPT, thereby attempting to enhance the tool’s outputs and align it more closely with their academic expectations. On the other hand, they adopted avoidance strategies to sidestep potential negative outcomes, such as avoiding tasks that could result in academic misconduct or focusing on maintaining academic integrity by rigorously checking AI-generated content. These strategies allowed students to reconcile the cognitive gap between their positive expectations of ChatGPT’s capabilities and the negative experiences of its actual limitations.

However, while cognitive dissonance theory traditionally emphasizes individuals’ efforts to reduce the gap between conflicting cognitions and behaviors, it does not sufficiently address the underlying factors that cause such dissonance to emerge in the first place. One of the key contributions of this study lies in extending Festinger’s (1957) original theory by elucidating the pathways that lead to cognitive dissonance in the context of ChatGPT use. Specifically, we identified that dissonance is driven by a tension between external pressures and expectations (such as subjective norms and technological expectancy) and barriers to effective usage (such as low tool efficacy and perceived risk). On the one hand, our findings suggest that cognitive dissonance functions as a consequence of using ChatGPT—emerging from mismatches between expectations and actual tool performance. On the other hand, as students experience value conflicts or emotional unease during tool use, they are intrinsically motivated to reduce this dissonance, often through various coping strategies. These strategies—such as optimizing prompt design or deliberately avoiding misuse—can in turn reshape students’ perceptions of ChatGPT’s efficacy and risks. These evolving perceptions may subsequently influence both future adoption behaviors and the intensity of dissonance itself (see Figure 1), forming a dynamic feedback loop between cognition, emotion, and behavioral intention.

In this sense, dissonance serves as a missing link in mainstream technology adoption models such as TAM and TPB, which primarily emphasize rational evaluations of usefulness and intention. By highlighting the emotional discomfort that arises from value misalignment, cognitive dissonance theory offers a unique lens to explain users’ ambivalence, hesitation, or even rejection of AI tools despite positive appraisals of their functionality. This insight complements the behavioral focus of TAM and TPB by addressing the affective conflicts that influence technology use in high-stakes academic settings.

## 5 Conclusion

This study explores the cognitive dissonance experienced by master’s students when using ChatGPT to assist in thesis writing and their coping strategies. Our findings indicate that the cognitive dissonance among master’s students primarily stems from the conflict between a strong intention to use the tool—driven by subjective norms and high technological expectancy—and the actual diverse options they have. This conflict between strong usage intention and abandonment behavior results in cognitive dissonance. To alleviate this dissonance, students adopted strategies such as improving prompt quality, feeding relevant field data, avoiding academic misconduct, and maintaining academic integrity, which effectively enhanced the efficacy of ChatGPT and reduced perceived risk.

Theoretically, this study makes significant extensions and additions to the Technology Acceptance Model and the Theory of Planned Behavior. Our findings suggest that not only does the perceived usefulness of current technology significantly influence usage intentions, but also the positive expectancy for the technology’s future development play a crucial role, enriching the concept of perceived usefulness in the TAM model. Additionally, we found that subjective norms play a key role in enhancing technological expectancy, indicating that combining TAM and TPB provides a more comprehensive explanation of users’ technology usage intentions. Furthermore, our research suggests that perceived behavioral control should be considered an important variable affecting attitudes, rather than merely a direct predictor of behavioral intentions. Notably, TAM and TPB frameworks tend to explain why users accept a particular technology or engage in a particular behavior, whereas, in reality, users may sometimes partially accept or even completely abandon a technology. Such behavior is difficult to fully explain within the existing TAM and TPB frameworks. Therefore, introducing cognitive dissonance theory can better explain the psychological mechanisms behind partial acceptance or rejection of technology. On the other hand, by incorporating TAM and TPB frameworks, this study contributes to the extension of cognitive dissonance theory by identifying and emphasizing the pathways and underlying causes that lead to dissonance, rather than focusing solely on the traditional understanding of individuals striving for consistency between cognition and behavior.

In light of our findings, we propose a set of recommendations for universities, supervisors, and educational technology companies to promote responsible and effective use of AI-assisted writing tools like ChatGPT in graduate education. Given the unstoppable trend of generative artificial intelligence, universities and graduate supervisors should not view ChatGPT as a dangerous tool and prohibit its use by master’s students. Instead, they should actively support, guide, and regulate the use of ChatGPT by master’s students in writing their theses. Universities can establish clear policies and guidelines to ensure that graduates adhere to academic integrity when using ChatGPT. They can also provide training and courses to teach students how to craft high-quality prompts, feed ChatGPT with relevant research materials, and verify the accuracy of generated content. Graduate supervisors can introduce the use of ChatGPT and best practices through workshops or seminars and provide timely feedback and guidance during their supervisees’ writing process. This approach not only encourages responsible and effective use of ChatGPT but also helps master’s students navigate the cognitive dissonance they may experience, fostering critical thinking and independent writing skills. To ensure this responsible use, it is essential to distinguish between using ChatGPT as a supportive tool—for language polishing, idea stimulation, or productivity enhancement—and relying on it as a substitute for critical engagement or original thinking. This distinction helps students benefit from AI assistance without compromising academic development or integrity. By guiding students in the ethical use of AI tools, universities and supervisors can support their academic growth while minimizing the psychological conflicts associated with integrating such technologies into their work. In addition, teachers should encourage students to explore diverse tools, including recommending and using localized generative AI tools like DeepSeek. At the same time, it should be acknowledged that generative AI tools are far from perfect. Therefore, for providers of large language model services, it is crucial to continuously optimize and improve these tools to better meet user needs. Educational technology companies and developers should pay attention to user feedback, enhance generative AI’s capabilities in deep analysis and the use of specialized terminology, build user trust, and lower the barrier to entry, making the tool easier to operate and use.

## 6 Limitations

This study was conducted in a context where access to ChatGPT was restricted. Due to legal and technical constraints in China, students often had to rely on VPNs and lacked official institutional support, which may have amplified their psychological tension and dissonance. However, the findings of this research may primarily apply to countries with limited access to ChatGPT, such as North Korea, China, Iran, Russia, and others. In contrast, students in Western or other open-access contexts may experience cognitive dissonance differently, with fewer external barriers and more institutional guidance. Future research could investigate the proposed model and its relevance in regions where ChatGPT is readily accessible. Comparing these contexts would help determine which aspects of cognitive dissonance are culturally specific and which are potentially universal, thereby offering a more nuanced and globally informed understanding of students’ psychological responses to AI-assisted writing.

## Data Availability

The raw data supporting the conclusions of this article will be made available by the authors, without undue reservation.
